# Editorial: Plant proteolytic enzymes: contributions and challenges to improve food availability against climate change effects

**DOI:** 10.3389/fpls.2024.1398867

**Published:** 2024-04-09

**Authors:** María Gabriela Guevara, Miguel A. Mazorra-Manzano, Dipak Gayen, Andreia Figueiredo

**Affiliations:** ^1^Biological Research Institute, National Council of Scientific and Technique Research (CONICET), University of Mar del Plata, Mar del Plata, Argentina; ^2^Centro de Investigación en Alimentación y Desarrollo (CIAD), Hermosillo, Sonora, Mexico; ^3^Department of Biochemistry, School of Life Sciences, Central University of Rajasthan, Rajasthan, India; ^4^Grapevine Pathogen Systems Lab, Biosystems & Integrative Sciences Institute, Science Faculty of Lisbon University, Lisbon, Portugal

**Keywords:** global nutrition, plant diseases, plant tolerance, plant biotechnology and breeding, plant proteases, plant protease inhibitor

Proteases play a ubiquitous role in both prokaryotic and eukaryotic organisms. Within plants, these enzymes serve as crucial regulators in a multitude of physiological processes, influencing protein homeostasis, organelle development, senescence, seed germination, protein processing, environmental stress response, and programmed cell death. A primary function of proteases involves the breakdown of peptide bonds, leading to irreversible post-translational modifications of proteins. They also function as signaling molecules, finely tuning cellular activities by cleaving and activating receptor molecules. Peptides generated through proteolysis play a pivotal role in regulating reactive oxygen species (ROS) signaling during oxidative stress in plants. Additionally, proteases contribute to cellular repair mechanisms by degrading misfolded and abnormal proteins into amino acids. This process not only aids in cell damage repair but also modulates biological responses to environmental stress. Proteases also play a key role in the biogenesis of phytohormones, which govern plant growth, development, and responses to environmental challenges (Moloi and Ngara, 2023).

Modern agriculture struggles to meet the increasing food, fodder, and raw material demands due to climate change and rapid population growth. Climate change is a major factor that negatively impacts crop yield potentials. Unlike animals, plants do not possess physical mobility or an adaptive immune system with mobile defender cells, and thus they have strategies to adapt and acclimate to changing environmental conditions by activating different protective mechanisms that trigger physiological, morphological, and biochemical changes. Inside the plant defense biochemical mechanism, proteolytic enzymes are the key regulators of several physiological processes, including environmental stress response. Therefore, the knowledge about the action mechanisms of these enzymes can be especially useful for enhancing plants’ environmental stress- tolerance. This Research Topic aimed at gathering novel research findings on the role of molecular proteolytic enzymes in the tolerance of plants to climate change effects. Five research articles were published covering several aspects of proteases research, from tool development for protease characterization (Duan et al.; Coppola et al.) to proteases role in seed development and nutritional value (Zheng et al.; Vorster et al.) and proteases participation in host defense mechanism and pathogen infection strategies (Backer et al.).

Regarding seed development, Zheng et al. examined the role of subtilisin-like proteases (SBT) in *Oryza sativa* (rice) caryopsis using a combination of bioinformatics, comparative genomics, and molecular approaches. Rice ranks among the top three global food crops, with human consumption representing 85% of its total production. Despite its crucial role in addressing hunger, rice production consumes a substantial 75% of the world’s total irrigated water resources. To enhance the sustainability of its irrigation system and promote water-saving production, various strategies have been implemented. However, a significant challenge associated with these cultivation methods is the suboptimal grain filling characterized by a shortened grain-filling period and reduced grain weight. This study highlighted tandem duplication and purifying selection as potential drivers for SBT expansion, pinpointing that domesticated rice presents more SBT genes than their wild relatives. The authors have also shown that four SBT genes were expressed specifically in caryopses on the proximal primary and secondary branches through presenting a putative role in caryopsis quality and phenotype of cultivated rice. Vorster et al. also provided an overview on the current knowledge of seed storage proteins, their degradation as well as on the serine protease inhibitors (SPI) system in seeds and what is known about the consequences when this system is modified. This perspective article includes the identification of seed specific SPIs; screening of germplasms, to identify plants with low seed inhibitor content, establishing serine protease-SPI ratios and lastly a focus on molecular techniques that can be used to modify seed SPI activity.

Within novel tools developed for protease characterization, the study by Duan et al. explored a new strategy to classify the A1 family of aspartic or acid proteases (APs), using a combination of phylogenetic and structure prediction based on AlphaFold (Jumper et al., 2021), a neural network-based model. APs have been classified into several groups based on the hierarchical and structural features of amino acid sequences (Rawlings et al., 2017). Among those, the A1 family has a higher number of members with myriad roles in physiological functions acting as regulators and signaling molecules for enhancing environmental stress tolerance (Sharma and Gayen, 2021). This study found a strategy more robust for understanding the function and evolution of a complex gene family of APs in plants like the A1 family in the plant model *Arabidopsis thaliana.* The review article by Coppola et al. highlighted the structural features and enzymatic activity of plant cathepsin B, emphasizing the discriminating feature between plant and animal isoforms. The article delves into the maturation process, subcellular localization, and provides a comprehensive account of the pivotal role played by cathepsin B in crucial plant physiological processes, such as programmed cell death (PCD), hypersensitive response (HR), early senescence, germination, seedling establishment, and leaf development. This knowledge shed light into our understanding of cellular signaling pathways involving cathepsin B, serving as a foundational prerequisite for applied advancements in plant biotechnology.

Plant disease outbreaks globally jeopardize food security and environmental sustainability, causing a decline in primary productivity and biodiversity. This negatively affects the environmental and socio-economic conditions of affected regions. Moreover, climate change exacerbates the outbreak risks by changing pathogen evolution and host-pathogen interactions, leading to the emergence of new and potentially harmful pathogenic strains. In this context, Backer et al. amazed the role of proteases in plant immunity, using dual RNA sequencing to investigate the transcriptomic responses of two *Persea americana* (avocado) rootstocks—Dusa^®^ (tolerant) and R0.12 (susceptible)—to infection by *Phytophthora cinnamomi.* Eighty-three differentially modulated host extracellular proteases were identified. The study suggests that the increased expression of subtilases could disrupt the biotrophic phase of *P. cinnamomi*, potentially due to subtilases’ role in establishing hypersensitive response. The study also reported that pathogen secreted protease inhibitors were major features in the incompatible interaction. It was hypothesized that an arms race between host proteases and pathogen protease inhibitors could be occurring. These results improve the knowledge of the interactions between pathogens and plants that are needed to develop climate-resilient crops.

Overall, these studies provide important contributions that aim to identify and characterize proteolytic enzymes, protease inhibitors and their roles in the plant defense mechanisms against the climate change effects, to elucidate valuable information for the development of stress-resilient crops with higher yield potentials.

## Author contributions

MG: Writing – original draft, Writing – review & editing. MM-M: Writing – original draft. DG: Writing – original draft. AF: Writing – original draft, Writing – review & editing.
